# Performance of two multiplex flow cytometric assays for antibody detection in Egyptian patients

**DOI:** 10.4102/ajlm.v12i1.2099

**Published:** 2023-05-18

**Authors:** Alshymaa A. Ahmed, Alia A. El Shahawy, Heba M. Kadry, Nora M. Said

**Affiliations:** 1 Department of Clinical Pathology, Faculty of Medicine, Zagazig University, Zagazig City, Egypt; 2 Department of Medical Microbiology and Immunology, Faculty of Medicine, Zagazig University, Zagazig City, Egypt

**Keywords:** autoantibodies, anti-double-stranded DNA, anti-neutrophil cytoplasmic antibodies, multiplex, performance

## Abstract

**Background:**

Autoantibodies are vital biomarkers for the diagnosis, assessment and prognostic determination of various autoimmune disorders.

**Objective:**

This study aimed to evaluate the performance of the two AtheNA Multi-Lyte^®^ systems for the detection of various autoantibodies.

**Methods:**

A total of 105 systemic lupus erythematosus patients, 35 patients with other autoimmune diseases (diseased controls), and 30 healthy volunteers (healthy controls) at Zagazig University Hospitals, Zagazig city, Al Sharqia governorate were tested for anti-double-stranded DNA (anti-dsDNA) antibodies using indirect immunofluorescence (IIF) and the AtheNA Multi-Lyte^®^ anti-nuclear antibodies-II system between May 2020 and April 2022. Seventy-five patients with clinically suspected autoimmune vasculitis (AIV) and 25 healthy volunteers were also tested for anti-myeloperoxidase and anti-proteinase 3 antibodies using IIF, the AtheNA Multi-Lyte^®^ AIV system, and enzyme-linked immunosorbent assay (ELISA).

**Results:**

The AtheNA anti-dsDNA test (98.5%) was more specific than IIF (96.9%) for diagnosing systemic lupus erythematosus, but both tests had the same sensitivity (38.1%). Combining both methods increased sensitivity to 47.6%, while increasing the cut-off of the AtheNA anti-dsDNA test to 134 international units/mL increased specificity to 100%. The AtheNA Multi-Lyte AIV system exhibited substantial agreement with IIF regarding anti-myeloperoxidase testing (κ = 0.65) and almost perfect agreement with ELISA (κ = 0.85). The AtheNA Multi-Lyte^®^ AIV system exhibited perfect agreement with IIF (κ = 1) and substantial agreement with ELISA for anti-proteinase 3 testing (κ = 0.63).

**Conclusion:**

AtheNA Multi-Lyte^®^ systems appear to be reliable for anti-dsDNA, anti-myeloperoxidase, and anti-proteinase 3 screening and may be an optimal choice for monitoring anti-dsDNA levels.

**What this study adds:**

It is necessary to evaluate various autoantibodies detection assays to increase both sensitivity and specificity of autoimmune diseases diagnostic approaches. AtheNA Multi-Lyte^®^ systems appear to be reliable for anti-dsDNA, anti-myeloperoxidase, and anti-proteinase 3 screening and may be an optimal choice for monitoring anti-dsDNA levels.

## Introduction

Systemic lupus erythematosus (SLE) and systemic vasculitis are multisystem autoimmune diseases characterised by the generation of circulating autoantibodies specific to different target antigens. The incidence and prevalence of both SLE and anti-neutrophil cytoplasmic antibody (ANCA)-associated vasculitis (AAV) show wide geographical variations.^1^ The overall incidence of SLE ranges from 0.3 cases per 100 000 population per year in Ukraine to 31.5 per 100 000 per year among Afro-Caribbean people living in the United Kingdom.^2^ In Egypt, the estimated prevalence of adult SLE is 6.1 cases per 100 000 persons, with a higher prevalence (11.3 per 100 000) reported among women.^3^ Anti-neutrophil cytoplasmic antibody-associated vasculitis inflicts considerable global effects, including elevated mortality rates, poor quality of life, and socio-economic burdens,^4^ and studies have demonstrated an AAV prevalence of between 48 and 184 cases per million individuals.^1^ In Egypt, AAV constitutes about 3.6% of vasculitides.^5,6^ However, there is inadequate data to identify the prevalence of AAV in Africa and South Asia.^7^

Autoantibodies are crucial biomarkers that guide the diagnosis, treatment, and follow-up of autoimmune disorders.^8^ New automated high-throughput assays are continuously being developed to replace conventional assays for detecting these autoantibodies. However, an accurate evaluation of these new techniques is mandatory to ensure that they are clinically valuable and can improve various medical decisions.^9^ According to the guidelines provided by the European League Against Rheumatism and the American College of Rheumatology, patients are classified as positive for SLE if they have serum anti-nuclear antibody titres ≥ 1:80.^10^ The presence of antibodies against double-stranded DNA (anti-dsDNA) is the serological hallmark for a diagnosis of SLE. An increased anti-dsDNA titre in association with low levels of the complement components C1q, C3, and C4 is indicative of an acute SLE exacerbation.^11,12^

Diverse methods have been developed for the detection of anti-dsDNA. The *Crithidia luciliae* immunofluorescence test is remarkably specific and yields a high positive predictive value for SLE. However, the sensitivity of *C. luciliae* immunofluorescence test for SLE diagnosis is low (ranging from 20% to 55%, depending on the kit supplier), and being a semi-quantitative test, it is not an optimal choice for clinical follow-ups and disease flare predictions.^13,14,15^ In contrast, the enzyme-linked immunosorbent assay (ELISA) is more sensitive (> 60%) than *C. luciliae* immunofluorescence test but has a lower specificity for SLE.^15,16^

Multiplex bead-based assays provide the advantage of assessing multiple antibody specificities simultaneously in small volumes of serum. One such technique is the addressable laser bead immunoassay, as exemplified by the commercially available Luminex^TM^ platforms. The sensitivity of addressable laser bead immunoassay is similar to that of ELISA for the detection of anti-dsDNA, while the specificity is comparable to that of *C. luciliae* immunofluorescence test.^17^ Existing guidelines for the diagnosis and follow-up of SLE such as ‘The 2019 European League Against Rheumatism and the American College of Rheumatology Classification Criteria for Systemic Lupus Erythematosus’^18^ do not specify a certain assay for anti-dsDNA detection. However, the guidelines advise that positive results should be confirmed by indirect immunofluorescence (IIF) or a Farr assay.^15,19^

The detection of ANCAs is vital for the diagnosis of the unique group of small-vessel vasculitic disorders named AAV. This group includes three main diseases: granulomatosis with polyangiitis, microscopic polyangiitis, and eosinophilic granulomatosis with polyangiitis. Patients with granulomatosis with polyangiitis are mainly proteinase 3-ANCA positive, while those with microscopic polyangiitis and eosinophilic granulomatosis with polyangiitis are mainly myeloperoxidase-ANCA positive.^20,21^ Indirect immunofluorescence is used as a screening test for ANCA in accordance with the 1999 International Recommendations for ANCA detection.^22^ This guideline advises the use of an antigen-specific assay to confirm a positive IIF test. However, the revised 2017 International Consensus of ANCA testing^23^ stated that the combined use of both IIF and antigen-specific immunoassays is not necessary. Specifically, a high-quality antigen-specific immunoassay is sufficient and does not need IIF confirmation.^24,25^

International guidelines and recommendations have emphasised the roles of anti-dsDNA and ANCA in the diagnosis and follow-up of autoimmune rheumatic diseases and vasculitic disorders.^15,23^ With this study, therefore, we aimed to assess the performance of the AtheNA Multi-Lyte^®^ test systems for the detection of these antibodies.

## Methods

### Ethical considerations

Ethical approval for this study was obtained from the Zagazig University-Institutional Research Board (ZU-IRB) (approval number: 5926). Written informed consent was obtained from participants and parents or guardians of patients younger than 18 years. All procedures were performed according to the principles of the Declaration of Helsinki.^26^ Patients’ files and samples were de-identified and coded, and all laboratory members were blinded to the participants’ data.

### Study population

This study was conducted in the Clinical Pathology Department of the Faculty of Medicine at Zagazig University, Zagazig, Al Sharqia, Egypt, from May 2020 to April 2022. All participants were of the same ethnicity. The required sample sizes were calculated using the OpenEpi program version 3 (https://www.openepi.com/SampleSize/SSCC.htm) at 95% confidence, 90% power, and a 10% dropout rate.^27^ We assumed the anti-dsDNA sensitivity in SLE patients to be 33% and that up to 3% of healthy controls and 6% of diseased controls can be anti-dsDNA positive.^15^ We also assumed an ANCA sensitivity of 46% in vasculitis patients and that up to 6% of healthy controls may be ANCA positive.^28^

Sera for anti-dsDNA testing were collected from 170 subjects, including 105 patients who met the revised criteria (European League Against Rheumatism and the American College of Rheumatology) for SLE classification^18^ and 65 control subjects matched for age and gender. The control subjects included 35 patients diagnosed with autoimmune diseases other than SLE (diseased controls) and 30 healthy participants (healthy controls). Sera for ANCA testing were collected from 75 patients with clinically suspected autoimmune vasculitis (AIV) and 25 healthy volunteers matched for age and gender (control group). Individuals who were pregnant, had a severe infection or systemic disease,^29^ or refused to sign the informed consent were excluded from the study. Personal data (age, gender, ethnicity) and medical data were collected from patients’ files retrieved from the Rheumatology Department after obtaining the required permissions.

For the diseased control group, the disease diagnosis was confirmed clinically and by the presence of specific autoantibodies. Serum rheumatoid factor and anti-CCP measured using the Cobas 6000 autoanalyzer (Roche Diagnostics, Forrenstrasse, Rotkreuz, Switzerland) were used to confirm rheumatoid arthritis. Using the anti-nuclear antibodies profile 3 immunoglobulin G (IgG) Euroline immunoblot assay (Euroimmun, Seekamp, Lübeck, Germany), we measured anti-Sjögren’s-syndrome-related antigen A and anti-Sjögren’s-syndrome-related antigen B for primary Sjögrens’ syndrome diagnosis, anti-SCL70 for systemic sclerosis diagnosis, and anti-Jo1 and anti-PM/SCL100 for polymyositis diagnosis.

### Sample collection

Two millilitres of whole blood were collected from each patient at the time of diagnosis. The blood samples were left to clot at room temperature for 15 min – 30 min and then centrifuged at 1000−2000 × g for 10 min. Sera were separated and stored at −80 °C prior to use.

### Multiplex microbead-based immunoassay

The AtheNA Multi-Lyte ANA-II Plus Test System (ZEUS scientific, Evans Way, Branchburg, New Jersey, United States) was used for the quantitative detection of IgG-class anti-dsDNA, the qualitative detection of IgG-class anti-nuclear antibodies, and the semi-quantitative detection of IgG-class antibodies specific for eight different nuclear antigens (Sjögren’s-syndrome-related antigen A, Sjögren’s-syndrome-related antigen B, Sm, RNP, Scl-70, Jo-1, Centromere B, and Histone) in the sera of enrolled subjects. The AtheNA Multi-Lyte AIV Plus Test System (ZEUS scientific, Evans Way, Branchburg, New Jersey, United States) was used for the semi-quantitative and qualitative detection of IgG-class antibodies specific for three analytes (myeloperoxidase, proteinase 3, and glomerular basement membrane) in the sera of enrolled subjects. Results > 120 international units/mL were considered positive. The microsphere suspension was prepared according to the manufacturer’s instructions and analysed using the Luminex^200^ platform (A DiaSorin Company, Technology Boulevard, Austin, Texas, United States). All samples were run in duplicate.

### Indirect immunofluorescence assay

Anti-dsDNA was detected via IIF using NOVA Lite^®^ dsDNA *C. luciliae* kits (Inova Diagnostics, Inc., San Diego, California, United States). The screening was performed at a serum dilution of 1/10, after which positive samples were diluted in the range of 1/20 to 1/640 for anti-dsDNA titre determination. Anti-neutrophil cytoplasmic antibody was detected via IIF using NOVA Lite^®^ ANCA Anti-neutrophil Cytoplasmic Autoantibody kits (Inova Diagnostics, Inc., San Diego, California, United States). The screening was performed at a serum dilution of 1/20. Both tests were performed according to the manufacturer’s instructions. Positive anti-myeloperoxidase appears as perinuclear staining (p-ANCA) while positive anti-proteinase 3 appears as cytoplasmic staining (c-ANCA).^30^

### Enzyme-linked immunosorbent assay for anti-myeloperoxidase and anti-proteinase 3

The ORGENTEC anti-myeloperoxidase (p-ANCA) and anti-proteinase 3 (c-ANCA) ELISA kits (Orgentec Diagnostika GmbH, Mainz, Germany) were used for the quantitative measurement of IgG-class autoantibodies specific for myeloperoxidase and proteinase 3. Results ≥ 5 IU/mL were considered positive. All samples were run in duplicate. We performed the assays and calculated the results according to the manufacturer’s instructions.

### Data analysis

The SPSS^®^ 20.0 software package (IBM Corporation, Armonk, New York, United States) was used for data processing and analysis. The overall detection results are expressed as percentages of the total number of samples. Agreement between different assays was assessed using Cohen’s kappa (κ) coefficient, with the following levels of agreement: 0.01–0.2 = slight agreement, 0.21–0.4 = fair agreement, 0.41–0.61 = moderate agreement, 0.61–0.8 = substantial agreement, and 0.81–1 = almost perfect or perfect agreement. Receiver operating characteristic curves were constructed, and the areas under the curves were calculated at 95% confidence intervals. The *T*-test and one-way analysis of variance with post hoc tests were used to compare ages, and the chi-square test was used to compare frequencies. The Spearman correlation coefficient test (*r*) was performed to examine the correlation between anti-dsDNA concentrations measured by AtheNA Multi-Lyte^®^ ANA-II (continuous variable) and anti-dsDNA titres estimated by IIF (ordinal variable).^31^ Results were considered significant at a *p*-value < 0.05.

Sensitivity, specificity, accuracy, and positive and negative predictive values were calculated using MedCalc (https://www.medcalc.org/) (MedCalc Software Ltd, Acacialaan, Ostend, Belgium).^32^ In addition to the clinical and analytical performance evaluations conducted, we compared the general characteristics of the assays used, including time taken for the run, estimated cost per test, and the technical experience required.

## Results

### General characteristics of patients

Anti-dsDNA antibodies were studied in 105 SLE patients and participants in control groups (30 healthy participants and 35 patients with autoimmune diseases other than SLE). Among patients with other autoimmune diseases, 18 had systemic sclerosis, 15 had rheumatoid arthritis, one had polymyositis, and one had primary Sjögren syndrome. Within the ANCA group, we examined 75 patients with AIV and 25 healthy participants matched for age and gender. Systemic lupus erythematosus patients’ ages ranged from 5 to 60 years (mean ± standard deviation: 32.4 ± 12.8 years), and 80 (76.1%) of them were women (Table 1). The ages of the AIV patients ranged from 14 to 77 years (mean ± standard deviation: 38.4 ± 15.7 years), and 50 (66.7%) of them were women.

**TABLE 1:** Demographic characteristics of SLE patients and control subjects at the Zagazig University Hospitals, Zagazig, Al Sharqia, Egypt, May 2020 – April 2022.

Demographic characteristics	SLE patients (*n* = 105)	Healthy controls (*n* = 30)	Diseased controls (*n* = 35)	*p*	AIV patients (*n* = 75)	Healthy controls (*n* = 25)	*p*
**Age (years)**	-	-	-	0.2	-	-	0.7
Mean ± s.d.	32.4 ± 12.8	32.3 ± 12.2	36.3 ± 13.6	-	38.4 ± 15.7	37.0 ± 13.1	-
Range	5–60	5–54	11–73	-	14–77	18–63	-
**Gender**	-	-	-	0.9	-	-	0.1
Female							
*n*	80	22	26	-	50	12	-
%	76.1	73.3	74.3	-	66.7	48	-
Male							
*n*	25	8	9	-	25	13	-
%	23.8	26.7	25.7	-	33.3	52	-

AIV, autoimmune vasculitis; s.d., standard deviation; SLE, systemic lupus erythematosus.

The most common symptom observed in the SLE group was skin manifestations (86.7%; 91/105) in the form of photosensitivity, malar, or discoid rash (Table 2). Articular manifestations in the form of arthritis and arthralgia were observed in 82.8% (87/105) of patients, while 75.2% (79/105) of patients had nephritis. Autoimmune vasculitis patients presented mainly with constitutional symptoms (80.0%; 60/75) and skin manifestations (57.3%; 43/75) in the form of skin ulcerations and palpable purpura. The lungs (66.7%; 50/75) and cardiovascular system (56.0%; 42/75) were the most frequently affected organs in these patients. Anti-nuclear antibodies were detected in all SLE patients (100%; 105/105) and 77.1% (27/35) of the diseased control group. Other autoantibodies were detected in both groups with variable frequencies.

**TABLE 2:** Clinical manifestations and serological findings of SLE patients, AIV patients, and diseased controls, Zagazig University Hospitals, Zagazig, Al Sharqia, Egypt, May 2020 – April 2022.

Clinical manifestations and serological findings	SLE patients (*n* = 105)		Diseased controls (*n* = 35)		AIV patients (*n* = 75)	
	*n*	%	*n*	%	*n*	%
**Clinical manifestations**						
Constitutional symptoms†	45	42.8	28	90.0	60	80.0
Skin manifestations‡	91	86.7	21	60.0	43	57.3
Articular manifestation§	87	82.8	20	57.1	33	44.0
Nephritis¶	79	75.2	10	28.6	37	49.3
Neurological††	15	14.2	12	34.2	20	26.7
Pulmonary‡‡	1	0.09	19	54.2	50	66.7
Haematological§§	65	61.9	4	11.4	37	49.3
Cardiovascular¶¶	2	1.9	11	31.4	42	56.0
Ear, nose, and throat†††	6	5.7	1	2.0	22	29.3
Eye‡‡‡	28	26.6	1	2.0	11	14.6
Raynaud’s phenomena	23	21.9	14	40.0	0	0.0
**Serological findings**						
ANA	105	100.0	27	77.1	N/A	-
SSA	32	30.5	5	14.3	N/A	-
SSB	41	39.1	2	4.0	N/A	-
SM	25	23.8	0	0.0	N/A	-
RNP	56	53.3	1	2.0	N/A	-
SCL70	12	11.4	15	42.8	N/A	-
JO1	5	4.8	1	2.0	N/A	-
Anti-centromere	3	2.9	4	11.4	N/A	-
Anti-histone	73	69.5	1	2.0	N/A	-

N/A, test not applied for this group of patients; ANA, anti-nuclear antibodies; SLE, systemic lupus erythematosus; AIV, autoimmune vasculitis; SSA, Sjögren’s-syndrome-related antigen A; SSB, Sjögren’s-syndrome-related antigen B; SM, Smith antigen; RNP, Ribonucleoprotein; SCL70, Scleroderma 70 or topoisomerase I; JO1, Histidyl-tRNA synthetase.

† , Fever, malaise, fatigue, weight loss.

‡ , Photosensitivity, malar rash, discoid rash, ulcerations, skin thickness, subcutaneous nodules, palpable purpura, digital necrosis.

§ , Arthritis, arthralgia, joint stiffness, joint deformities and limitation of movements.

¶ , Haematuria, proteinuria.

†† , Paraesthesia, headache, confusion.

‡‡ , Cough, wheezing, haemoptysis, dyspnoea.

§§ , Bleeding, thrombosis, purpura, ecchymosis.

¶¶ , Intermittent claudication of extremities, chest pain, decreased peripheral pulses.

††† , Epistaxis, sinusitis, hoarse voice.

‡‡‡ , Sicca syndrome, redness and pain.

### Anti-double-stranded DNA testing by IIF and AtheNA Multi-Lyte ANA-II test

Overall, 38.1% (40/105) of SLE patients were positive for anti-dsDNA by IIF or AtheNA Multi-Lyte^®^ ANA-II. Additionally, AtheNA Multi-Lyte^®^ ANA-II yielded equivocal results for 9.5% (10/105) of samples (anti-dsDNA 100 IU/mL – 120 IU/mL). All healthy controls were negative for anti-dsDNA using both IIF and AtheNA Multi-Lyte^®^ ANA-II. One patient with rheumatoid arthritis had positive anti-dsDNA results with IIF, and another one had positive results with both methods.

There was a moderate correlation between the anti-dsDNA concentrations measured using AtheNA Multi-Lyte ANA-II and the anti-dsDNA titres estimated using IIF (*r* = 0.55, *p* < 0.001). The AtheNA Multi-Lyte^®^ ANA-II and IIF results exhibited substantial agreement with respect to anti-dsDNA testing (κ = 0.66, 95% confidence intervals: 0.53–0.79, percent agreement: 87.6%, *p* < 0.001).

**FIGURE 1:**
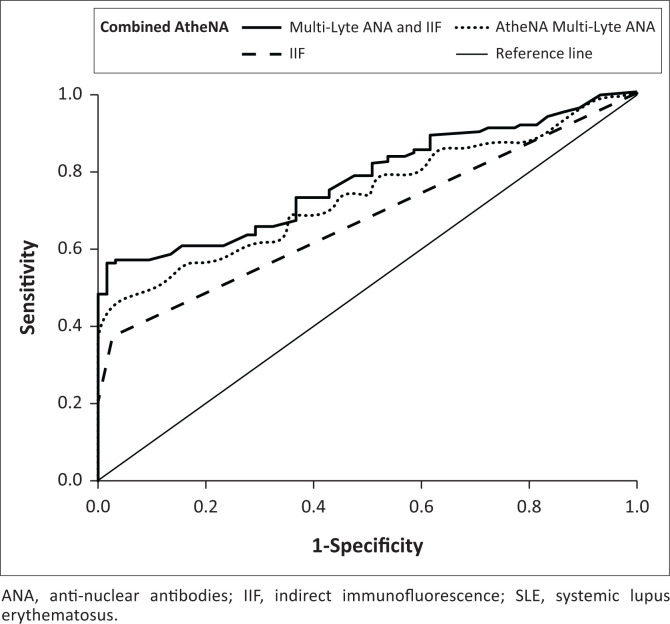
Receiver operating characteristic curves showing the clinical performance of IIF and AtheNA Multi-Lyte ANA-II when conducted separately and in combination for the diagnosis of SLE among patients attending Zagazig University Hospitals, Zagazig, Al Sharqia, Egypt, between May 2020 and April 2022. The European League Against Rheumatism and the American College of Rheumatology criteria was used as the gold standard.

At the manufacturer’s cut-off (120 IU/mL), the sensitivity of AtheNA Multi-Lyte ANA-II for SLE diagnosis was 38.1%, and the specificity was 98.5% (Online Supplementary Table 1, Figure 1). The best Youden’s index was obtained when the cut-off was lowered to 109 IU/mL, at which the sensitivity increased to 46.7% and the specificity decreased to 96.9%. Full (100%) specificity was reached by increasing the cut-off to 134 IU/mL, but this caused the sensitivity to decrease to 37.1%. For IIF, the sensitivity was 38.1% and the specificity was 96.9%. However, the combination of both methods at the manufacturer’s cut-off (120 IU/mL) increased the sensitivity to 47.6% and the specificity to 96.9%.

**FIGURE 2:**
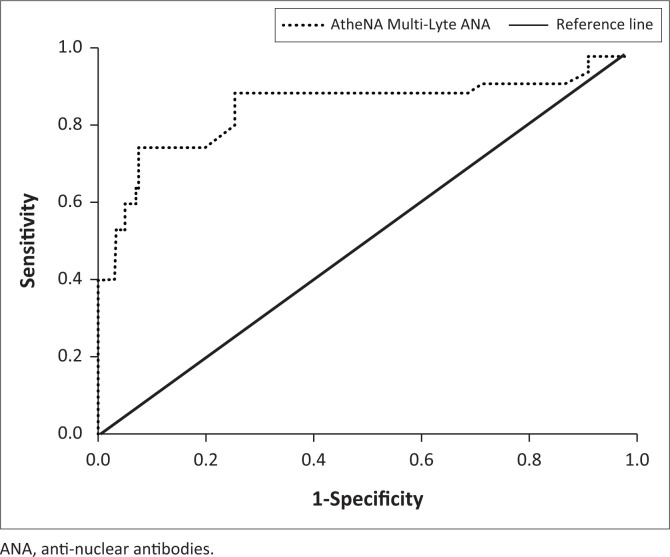
Receiver operating characteristic curve showing the analytical performance of AtheNA Multi-Lyte ANA-II in the detection of anti-double-stranded DNA among patients with SLE attending Zagazig University Hospitals, Zagazig, Al Sharqia, Egypt, between May 2020 and April 2022. Indirect immunofluorescence was used as the gold standard.

To determine its analytical performance, we compared the results of AtheNA Multi-Lyte^®^ ANA-II to those of IIF, the gold-standard method. AtheNA Multi-Lyte^®^ ANA-II had a sensitivity of 73.8% (95% confidence intervals: 57.96% – 86.14%), a specificity of 92.19% (86.10% – 96.19%), a positive predictive value of 75.61% (62.47% – 85.24%), a negative predictive value of 91.47% (86.56% – 94.70%), an accuracy of 87.65% (81.74% – 92.19%), and an area under the curves of 0.830 (0.746–0.914) (Figure 2).

### Anti-myeloperoxidase and anti-proteinase 3 analyses using IIF, AtheNA Multi-Lyte^®^ IIF, and ELISA

Overall, 62.7% (47/75) and 14.7% (11/75) of patients tested positive for p-ANCA and c-ANCA by IIF, while only 40.0% (30/75) and 14.7% (11/75) tested positive for anti-myeloperoxidase and anti-proteinase 3 by AtheNA Multi-Lyte AIV. Using the ELISA method, 32.0% (24/75) of patients were positive for ani-myeloperoxidase, and 28.0% (21/75) were positive for anti-proteinase 3. All healthy volunteers were negative for anti-myeloperoxidase and anti-proteinase 3 using all three methods. All subjects were negative for anti-glomerular basement membrane.

Regarding the results of anti-myeloperoxidase testing, we observed a substantial agreement between IIF and AtheNA Multi-Lyte AIV (κ = 0.653), a moderate agreement between IIF and ELISA (κ = 0.525), and an almost perfect agreement between AtheNA Multi-Lyte AIV and ELISA (κ = 0.853) (Table 3). For anti-proteinase 3 testing, there was a perfect agreement between AtheNA Multi-Lyte AIV and IIF (κ = 1), and a substantial agreement between ELISA and both IIF and AtheNA Multi-Lyte AIV (κ = 0.635). Moreover, the ELISA results were highly correlated with the AtheNA Multi-Lyte AIV results for the analyses of both anti-myeloperoxidase (*r* = 0.606, *p* < 0.001) and anti-proteinase 3 (*r* = 0.363, *p* = 0.006).

**TABLE 3:** Agreement between the studied methods (AtheNA Multi-Lyte AIV, IIF, and ELISA) for ANCA detection among AIV patients attending Zagazig University Hospitals, Zagazig, Al Sharqia, Egypt, between May 2020 and April 2022.

Anti-neutrophil cytoplasmic antibodies	Methods	Percent agreement (%)	Kappa	*p*
Anti-MPO	AtheNA Multi-Lyte AIV vs IIF	83	0.653 (0.51–0.79)	< 0.001
	ELISA vs IIF	77	0.525 (0.38–0.68)	< 0.001
	AtheNA Multi-Lyte AIV vs ELISA	94	0.853 (0.78–1.01)	< 0.001
Anti-proteinase 3	AtheNA Multi-Lyte AIV vs IIF	100	1	< 0.001
	ELISA vs IIF	90	0.635 (0.44–0.83)	< 0.001
	AtheNA Multi-Lyte AIV vs ELISA	90	0.635 (0.44–0.83)	< 0.001

Note: Kappa = Cohen’s kappa (κ) coefficient.

MPO, Myeloperoxidase; IIF, indirect immunofluorescence; ELISA, enzyme-linked immunosorbent assay; AIV, autoimmune vasculitis.

The clinical performances of the anti-myeloperoxidase and anti-proteinase 3 detection methods could not be assessed because a definitive diagnosis of AIV relies on histopathology, which was not performed routinely for all patients. However, we assessed the analytical performances of AtheNA Multi-Lyte AIV and ELISA versus IIF, the gold-standard method. For anti-myeloperoxidase, AtheNA Multi-Lyte AIV had a higher sensitivity than ELISA (63.8% vs 51.6%), while both tests had a specificity of 100% (Online Supplementary Table 2). For anti-proteinase 3, both methods had a sensitivity of 100%, while AtheNA Multi-Lyte AIV had a higher specificity than ELISA (100% vs 88.8%).

A comparison of the general characteristics of the methods used showed that IIF had the highest estimated cost (240.00 Egyptian Pounds [EGP]) for anti-dsDNA testing as further dilutions were required to obtain the antibody titre using IIF (Table 4). However, for ANCA testing, ELISA had the highest cost (200.00 EGP) and time consumption (180 min) due to the need for two separate kits to obtain antigen-specific identification of anti-myeloperoxidase and anti-proteinase 3. In addition to being a quantitative rather than semi-quantitative assay, the AtheNA Multi-Lyte also required the least amount of technical expertise (a bachelor’s degree in clinical laboratory science), was the least time-consuming (90 min), and had intermediate costs (120.00 EGP).

**TABLE 4:** Comparison of the general characteristics of AtheNA Multi-Lyte, IIF, and ELISA used for anti-dsDNA and ANCA detection among SLE and AIV patients attending Zagazig University Hospitals, Zagazig, Al Sharqia, Egypt, between May 2020 and April 2022.

Methods	Cost		Time	Technical experience requirement	Quantitative/qualitative
	anti-dsDNA	ANCA			
IIF	240.00 EGP	80.00 EGP	120 min	+++	Semi-quantitative
ELISA	N/A	200.00 EGP	180 min	+	Quantitative
AtheNA Multi-Lyte assays†	120.00 EGP	120.00 EGP	90 min	+	Quantitative

IIF, indirect immunofluorescence; EGP, Egyptian Pounds; ANCA, anti-neutrophil cytoplasmic antibodies; SLE, systemic lupus crythematosus; AIV, autoimmune vaculitis; ELISA, enzyme-linked immunosorbent assay.

+ , Bachelor’s degree in clinical laboratory science;

+++ , specialised immunologist degree.

† , AtheNA Multi-Lyte ANA-II assay was used for anti-dsDNA and AtheNA Multi-Lyte AIV assay was used for ANCA testing.

## Discussion

This work was designed to evaluate the performance of two commercially available multiplex bead-based flow cytometric assays, the AtheNA Multi-Lyte ANA-II and AtheNA Multi-Lyte AIV systems, for the detection of anti-dsDNA and ANCAs. The multiplex assays had reasonable performance compared with traditional methods used for the detection of autoantibodies.

For the detection of anti-dsDNA, our data demonstrated a substantial agreement between the AtheNA Multi-Lyte test system and IIF. However, we observed a highly significant correlation between anti-dsDNA concentration by AtheNA Multi-Lyte test system and IIF antibody titre. Our findings are in concordance with the results of previous studies conducted in Canada in 2010^33^ and 2013,^34^ and in Italy in 2018.^15^ These studies used a device from a different manufacturer (BioPlex 2200, Bio-Rad Laboratories, Hercules, California, United States) and reported the same level of agreement and correlation with disease activity as achieved with IIF. According to our data, the AtheNA Multi-Lyte test system had the same sensitivity as IIF for anti-dsDNA detection, as well as improved specificity. Moreover, the combination of both methods increased the sensitivity to 47.6%. Two studies performed by Infantino and colleagues in Italy in 2015^14^ and 2018^15^ reported that the multiplex bead-based assay was more sensitive than IIF but was slightly less specific.^15^

Based on our observations, the AtheNA Multi-Lyte test system for anti-dsDNA detection provides multiple advantages, as it is a simple and rapid method with a reasonable cost and has acceptable clinical and analytical performance. Although the combination of AtheNA Multi-Lyte ANA-II and IIF provided better sensitivity, economic factors might hinder the routine use of this combination for screening. Our findings favour the use of the AtheNA Multi-Lyte test for patient follow-ups because this one-step quantitative analysis is simpler than the multiple dilutions required for the semi-quantitative IIF determination of antibody titres.

The AtheNA Multi-Lyte test systems exhibited almost perfect agreement with ELISA and moderate agreement with IIF with respect to anti-myeloperoxidase detection. Additionally, the AtheNA Multi-Lyte assay exhibited an almost perfect agreement with ELISA and a perfect agreement with IIF for anti-proteinase 3 detection. These results are consistent with the results of previous studies, including one performed in the Netherlands in 2007, and another multicentre study performed in 2017 that recruited patients from Germany, Denmark, the Netherlands, and Belgium.^35,36^ Only one study, conducted in the United States in 2018,^37^ reported a moderate agreement between a multiplex bead-based assay and ELISA for both anti-myeloperoxidase (κ = 0.35) and anti-proteinase 3 (κ = 0.53) detection. This difference might be attributable to the use of kits produced by a different manufacturer (Bio-Plex^®^ 2200 testing platform, Bio-Rad Laboratories, Hercules, California, United States). The analytical performance of AtheNA Multi-Lyte AIV was superior to that of ELISA for both anti-myeloperoxidase and anti-proteinase 3 detection. For anti-myeloperoxidase testing, AtheNA Multi-Lyte AIV was more sensitive than ELISA, while both methods were equally specific. For anti-proteinase 3 testing, both methods were equally sensitive, whereas AtheNA Multi-Lyte AIV was more specific.

Regarding ANCA detection, histopathology is the definitive diagnosis for AIV.^10^ However, these data were not available for the patients included in this study. Therefore, we were unable to assess the clinical performance of AtheNA Multi-Lyte AIV and ANCA IIF assays. Still, the AtheNA Multi-Lyte test systems provided good analytical performances and significant agreement with the results of conventional assays, warranting their use for anti-myeloperoxidase and anti-proteinase 3 screening.

### Limitations

This was a single-centre study with a relatively small sample size. The study was also limited by a lack of histopathology data for patients with suspected AIV.

### Conclusion

The AtheNA Multi-Lyte test systems are reliable and robust for the simultaneous detection of autoantibodies. These tests aid in the diagnosis and follow-up of systemic rheumatic disorders and the diagnosis of vasculitic disorders. Multiplex autoantibody testing is less time-consuming than traditional single-antibody assessment and requires only small sample volumes. Future studies that include the definitive diagnosis of AIV according to international standards are required to yield a better understanding of the clinical performances of anti-myeloperoxidase and anti-proteinase 3 tests. We recommend the conduct of a multicentre study with a larger sample size to confirm our findings.
